# Cross-Sector Partnerships for Systemic Change: Systematized Literature Review and Agenda for Further Research

**DOI:** 10.1007/s10551-018-3922-2

**Published:** 2018-06-01

**Authors:** Amelia Clarke, Andrew Crane

**Affiliations:** 1 Master of Environment and Business Program, School of Environment, Enterprise and Development, University of Waterloo, Room 4229, Environment 3 Building, 200 University Ave. West, Waterloo, ON N2L 3G1 Canada; 2 Centre for Business, Organisations and Society (CBOS), School of Management, University of Bath, Bath, BA2 7AY UK

**Keywords:** Systemic change, Transformation, Partnership

## Abstract

The literature on cross-sector partnerships has increasingly focused attention on broader systemic or system-level change. However, research to date has been partial and fragmented, and the very idea of systemic change remains conceptually underdeveloped. In this article, we seek to better understand what is meant by systemic change in the context of cross-sector partnerships and use this as a basis to discuss the contributions to the Thematic Symposium. We present evidence from a broad, multidisciplinary systematized review of the extant literature, develop an original definition of systemic change, and offer a framework for understanding the interactions between actors, partnerships, systemic change, and issues. We conclude with some suggestions for future research that we believe will enhance the literature in its next phase of development.

## Introduction

Cross-sector partnerships—by which we mean relatively intensive, long-term interactions between organizations from at least two sectors (business, government, and/or civil society) aimed at addressing a social or environmental problem—are now a fixture in management research and practice. They have become a central theme in research about the social role and responsibilities of business (Seitanidi and Crane 2009, 2014), the emergence and effectiveness of new forms of private governance (Auld et al. 2015; Cashore et al. 2004; Crane 2011; Hahn and Pinkse 2014; Pattberg 2005), and the shifting practices, performance, and legitimacy of civil society (Baur and Palazzo 2011; Baur and Schmitz 2012; Dauvergne and LeBaron 2014; Herlin 2015).

The broad interdisciplinary literature that has subsequently emerged around cross-sector partnerships has addressed a range of conceptual, empirical, practical, and methodological issues. These have been comprehensively mapped in a series of systematic reviews of the cross-sector partnerships literature including those by Bryson et al. (2006, 2015), Gray and Stites (2013), Branzei and Le Ber (2013), Laasonen et al. (2012), Austin and Seitanidi (2012a, b), and Selsky and Parker (2005).

A key theme in this literature has been an examination of the performance or effectiveness of cross-sector partnerships, particularly with respect to achieving specific organizational and societal goals and having meaningful impact on supposed beneficiaries (Clarke and Fuller 2010; Clarke and MacDonald 2016; Seitanidi et al. 2010; Van Tulder et al. 2015). A range of issues have been explored within this stream of the literature from analyses of different types, metrics, or meanings of performance, to exploring the managerial challenges of aligning or accommodating divergent goals among partners, and the challenges of devising effective assessment methodologies.

Given that one of the key drivers of partnerships is the need to address complex social and environmental problems that are too large or intractable for one organization or sector to tackle alone (Waddock 1989), much attention has focused on partnership performance at a macro-level. That is, rather than seeing partnerships simply in terms of organization-level outcomes, scholars have increasingly focused attention on broader systemic or system-level change. For example, Senge et al. (2006, pp. 421–422) discuss the “growing severity of systemic issues” and the realization that “eradicating the systemic causes of poverty was not going to happen through NGO and governmental actions alone” as key drivers for business and civil society actors to “overcome the reluctance to enter into deeper working relationships”. Likewise, Austin and Seitanidi (2012a, p. 952) in their review of the outcomes of cross-sector partnerships state that “at a broader societal level the collaboration may also contribute to welfare-enhancing systemic change in institutional arrangements, sectoral relationships, societal values and priorities, and social service and product innovations, as well as improving the environment with multiple societal benefits”.

The interest in systemic change as a potential outcome of cross-sector partnerships speaks both to the enthusiasm among researchers and practitioners for exploring the potential of partnerships to effect deeper-level impact on the social and environmental systems in which partners are embedded, and to the unease among critics regarding the negative effects such transformations might wreak. Extant research, for example, has explored the ways in which cross-sector initiatives can enhance the system-level governance of social and environmental problems (Cashore et al. 2004; Auld et al. 2015), while critics have pointed to the corporatization of activism (Dauvergne and LeBaron 2014) and societal imbalance (Mintzberg 2015) as adverse system-level problems that partnerships might contribute to.

Despite this growing attention to the role of cross-sector partnerships in systemic change, research has to date been partial and fragmented. To begin with, the very idea of systemic change remains underdeveloped in the literature. The question of what exactly is meant by “systemic change” in the context of cross-sector partnerships remains somewhat unclear, and has often been left unspecified in studies that invoke the term. Similarly, how (if at all) “systemic change” differs from other terms used in the literature such as “system change” (Selsky and Parker 2005), “transformative change” (Linnenluecke et al. 2017), and “institutional change” (Vurro and Dacin 2014) has yet to be established. To date, there seems to be little clarity or consistency in usage of the terms, and there appears to be little by way of common definitions across the literature. These problems are compounded when we look beyond the management literature to other disciplines such as politics, health, geography, development studies, and environmental science where the systemic effects of cross-sector partnerships have also been explored. In such disciplines, the types of systems under examination and the relevant conceptions of what constitutes “systemic change” potentially differ even more. Such ambiguity both within and beyond the management literature can lead to conceptual confusion and imprecision in theory development and testing, as well as inhibiting cross-disciplinary fertilization.

Beyond these important definitional problems, there is a host of further challenges that need addressing in exploring cross-sector partnerships for systemic change. However, these all rest on establishing a better conceptual foundation in terms of enhanced construct clarity. For instance, it has yet to be determined how to design effective partnerships for achieving systemic change, or perhaps more critically, under what conditions they can achieve such change and under which they cannot. To answer such question though requires that we are clear on what kind of system we are talking about, and what form of systemic change we are concerned with. Likewise, the critical question of how to balance or coordinate system-level changes with more micro- and meso-level changes (or even other macro-level changes) is dependent on refining our conception of systemic change and delineating it from other similar but different forms of change.

Our goal in this paper is therefore to understand better what is meant by systemic change in the context of cross-sector partnerships, and use this as a basis to discuss the contributions to this Thematic Symposium and to elaborate some potential pathways for further research. To do so, we present evidence from a broad, multidisciplinary review of the extant literature. We analyse this to explore some of the different ways that systemic change has been considered in cross-sector partnership, both explicitly and implicitly, and to develop a definition of systemic change and a framework of cross-sector partnerships for systemic change that can be used by future researchers to position their research in relation to competing approaches and definitions.

## Methods

To get a better sense of how systemic change has been dealt with to date in the cross-sector partnership literature, we conducted a systematized, interdisciplinary review. Systematized reviews include elements of a systematic review, but do not aim for complete comprehensiveness (Grant and Booth 2009). That is, we were looking to provide a structured, indicative review of a very broad and ill-defined literature base but without necessarily seeking to analyse everything that has been written on the subject of cross-sector partnerships for systemic change. We searched for relevant literature in three databases, namely Google Scholar, ProQuest, and Scopus. Each of the three databases offers different search functionality. To get the widest range of potential literature, we used the most inclusive search fields available for each database. Therefore, with Google Scholar we were only able to search by title, with ProQuest we could search by title and abstract, and ProQuest allowed the widest scope, offering search by title, abstract, and keywords. For convenience, we limited the search to journal articles only, but included all articles published up to and including August 2017.

With respect to search terms, we treated both “cross-sector partnership” and “systemic change” as non-exclusive labels delineating the relevant literature. We therefore used a number of synonyms and related terms across a series of searches, including those that we knew from experience were likely to be featured in disciplines beyond management. For cross-sector partnership, we searched “cross-sector partnership”, “public–private”, “business–NGO”, “collaborative governance”, and “collaborative planning”. When combined with “AND systemic change”, this resulted in 15 articles (including duplicates) across the three databases. Including “AND system change” yielded an additional 26 articles (including duplicates). Therefore, to generate a larger dataset we expanded beyond these two core terms to also include “system-wide change” (0 results), “major social change” (0 results), “institutional change” (84 results), and finally “transformation” (737 results).

The inclusion of the latter two terms clearly encapsulated a range of articles with little or no correspondence to systemic change, but they also yielded a number of studies that did indeed engage with ideas of change at a system level. A variety of “institutional change” studies, for example, explicitly considered institutional fields of interconnected organizations as a system, while some of the “transformation” studies considered transformational change in social and environmental issues in terms of systems. Altogether, the combined searches yielded 862 articles in total (including duplicates), heavily weighted towards the search term “transformation”. We then removed duplicates and made an initial assessment of relevance by reading the abstracts. This led to the removal of a large proportion of the “institutional change” and “transformation” articles. Following this, we selected the top 100 most relevant articles—i.e. those that explicitly appeared to deal with some kind of systemic change, broadly defined, through cross-sector interactions—as our corpus of literature to analyse.

### Approaches to Systemic Change in the Cross-Sector Partnership Literature (Results)

#### Overview of the Articles by Discipline

The 100 articles ranged from 1994 to 2017. The citation counts ranged from 0 to 406. Three of the four most cited articles use institutional change. Table 1 shows the summary of results by discipline, indicating the number of articles in each discipline, the search terms that resulted in those articles, and the sectors engaged in those articles.

**Table 1:** Summary of results

Discipline	# of articles	Search terms (some articles appeared in more than one search)	Sectors engaged
Anthropology/history	3	Transformation + public–private (2) Institutional change + public–private (1)	Public and private (2) Public and civil society (1)
Communications	2	Transformation + public–private (2)	Public and private (2)
Economics	5	Transformation + public–private (3) Transformation + cross-sector (2) Institutional change + public–private (1)	Public and private (4) Tri-sector (1)
Education	3	Transformation + public–private (3)	Public and private (2) Tri-sector (1)
Engineering	1	Transformation + public–private (1)	Public and private (1)
Environment/sustainability	8	Transformation + public–private (7) Transformation + cross-sector (1) System change + collaborative planning (1)	Public and private (5) Tri-sector (3)
Geography	5	Transformation + public–private (4) Institutional change + public–private (1)	Public and private (3) Tri-sector (2)
Health	10	Transformation + public–private (7) System change + public–private (2) Systemic change + public–private (1) Transformation + collaborative governance (1)	Public and private (7) Public and civil society (3)
Information technology	8	Transformation + public–private (6) Transformation + collaborative governance (2)	Public and private (6) Tri-sector (2)
Law	2	Institutional change + public–private (2)	Public and private (2)
Management	27	Transformation + public–private (21) Transformation + cross-sector (5) Institutional change + public–private (3) Transformation + collaborative governance (1) Transformation + collaborative planning (1) Systemic change + cross-sector (1) Systemic change + public–private (1)	Public and private (16) Private (5) Tri-sector (3) Private and civil society (2) Public and civil society (1)
Political science	6	Transformation + public–private (3) Institutional change + public–private (2) Transformation + cross-sector (1) Transformation + collaborative governance (1)	Public and private (3) Tri-sector (2) Public (1)
Public administration/public policy	12	Transformation + public–private (8) Institutional change + public–private (2) Institutional change + collaborative governance (2) Transformation + cross-sector (1) Transformation + collaborative governance (1)	Public and private (7) Tri-sector (2) Public and civil society (2) Public (1)
Social work/sociology	4	Transformation + public–private (2) Institutional change + public–private (1) Systemic change + public–private (1) System change + public–private (1)	Public and private (3) Public (1)
Urban planning	4	Institutional change + collaborative planning (2) Institutional change + public–private (1) Transformation + public–private (1)	Public and private (2) Public (2)
Totals	100	Transformation + public–private (70) Institutional change + public–private (14) Transformation + cross-sector (10) Transformation + collaborative governance (5) Systemic change + public–private (3) System change + public–private (3) Institutional change + collaborative governance (2) Institutional change + collaborative planning (2) Systemic change + cross-sector (1) Systemic change + collaborative planning (1) Transformation + collaborative planning (1)	Public and private (65) Tri-sector (16) Public and civil society (7) Public (5) Private (5) Private and civil society (2)

As can be seen from the table, our search found relevant articles in numerous disciplines. Management is the highest with 27 articles, followed by public administration/public policy, then health, and then environment/sustainability and information technology. Discipline was determined based on the title of the journal. In terms of search results, “transformation + public–private” is by far the highest, and it returned articles in almost every discipline (except law). Institutional change is also found in nine of the disciplines. Systemic or system change is only found in four disciplines (management, social work/sociology, health, and environment/sustainability). For the other synonyms, public–private is found in all disciplines, cross-sector is found in five disciplines, collaborative governance in four disciplines, and collaborative planning in three.

While articles were selected for matching the keywords, their content sometimes only focuses on one or two sectors. Articles that focus on both the private and public sectors are found in all disciplines. Tri-sector interactions are written about in eight disciplines, public and civil society in four, public in four, private and civil society in one, and private in one.

#### Overview of the Articles by Year

**Fig. 1:**
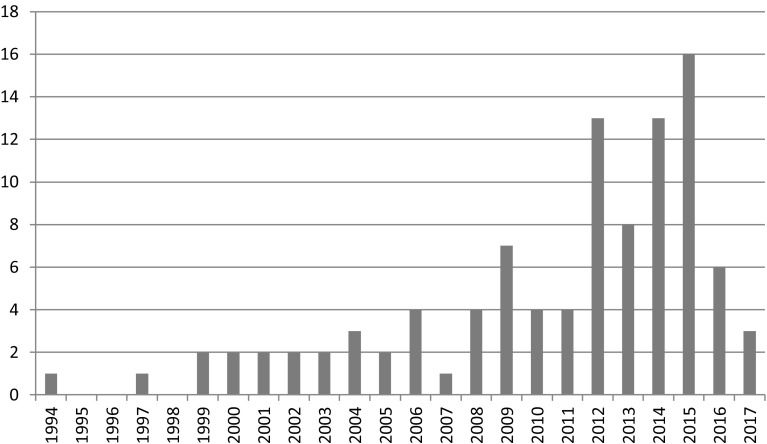
Number of articles per year

There is no particular pattern to the content over time that can be discerned from this sample, but as can be seen in Fig. 1 there are more papers in recent years. The oldest two articles use transformation and private–public. The two in 1999 use systemic change and system change. The first institutional change in the sample is from 2000.

#### Overview of the Articles by Systems and Scales

In terms of the scale of systems studied in these articles, 50 are focused at the national scale, 13 at the local scale, and six more at the sub-national (state/region/province) level. Eight more are at a multinational or global scale. These account for 75 of the 100 articles. There are four articles on multi-actor supply chains that focus on the transformation of a product/material (e.g. Azevedo et al. 2004; Kubde and Bansod 2010) and two focus on transformation of a specific organization (e.g. Wieters 2016). The remaining 17 articles had less obvious scales; for example, they consider institutional change in the context of partnerships (e.g. Matos-Castaño et al. 2014), or in the structure of an organizational field (e.g. Montgomery and Oliver 2009), or in a conceptual piece about new institutionalism (Ingram and Clay 2000).

At the national scale, the types of systems studied are diverse, including economic empowerment programme system in South Africa (Hamann et al. 2008), the wine sector in Argentina (McDermott et al. 2009), water sector in China (Lee 2010), the Food and Drug Administration’s regulation of markets in the USA (Frohlich 2012), the health system in Saudi Arabia (Alonazi 2017), the higher education systems in the UK (Hagen 2002), telecommunications policy in South Korea (Larson and Park 2014), e-government in Canada (Langford and Roy 2006). As can be seen from this sampling, cross-sector partnerships and interactions are studied in countries all over the world. The most frequent topic is health, with 11 articles at the national or sub-national level.

At the local scale, the systems include homeless services system (Mosley 2014), local public health delivery system (Ingram et al. 2012), water system or watershed (Gopakumar 2014; Weber 2009), urban development (Zhang et al. 2016), land management (McCauley and Murphy 2013), urban governance (Guarneros-Meza 2009; Meijer and Bolívar 2016). This is only a sampling of the articles which focus at the local scale, but shows the diversity of topics. Water is the most frequent topic area with eight articles focusing on this topic.

#### Types of Partnerships Considered

Given the search criteria, not all the articles are focused on cross-sector partnerships. Some are focused on cross-sector interactions more generally, including the boundaries of the public and private sectors (e.g. Ruane 1997). That said, the majority of those screened into the top 100 articles consider a collaboration or partnership, with the most frequent being around public–private partnerships (PPP). Of the 100 articles, 16 use the term public–private partnership (or PPP) in their title.

#### Definition of Systemic Change

Even with a specific search for articles on systemic change, there is no clear usage of the concept. Many articles use a term without ever defining it (e.g. Heikkila and Gerlak 2005). In fact, despite our best efforts, our review of the relevant literature did not reveal a single specific definition of systemic change that might be considered fit for the purpose of capturing the phenomenon in a comprehensive and precise way. We can, however, elucidate some general inferences about what is meant with respect to systemic change in the literature, and from these inferences build our own definition, which follows at the end of this section.

In general, the articles consider systemic change (or transformation or institutional change) in relation to the system under study. The actual transformation might be indicated by a significant change in an institutional field’s structure, in a policy, or in the system’s function. Here is a sampling of representative definitions for each of our search terms (institutional change, transformation, systemic/system change).

Institutional change articles are grounded in the core concepts of institutional theory and therefore consider change over time in an institutional field, or by an actor on an institutional field. This meaning of institutional change is exemplified by this quotation:

…questions remain on how the process of institutional change occurs in the context of PPPs, why and how different environments react differently to the same set of stimuli and how institutional environments affect and are in turn affected by the PPP projects that are undertaken. Answers to these questions could aid policy makers as they attempt to design and alter institutions to foster PPPs in their environments. (Matos-Castaño et al. 2014, p. 48)

From this quotation, it can be seen that the change being studied might be within the PPP as a result of the environment’s influence, or it might be within the environment as a result of the PPP’s influence. The focus in this article is not on the social, environmental, or economic problem to be solved through the PPP, but rather on the structural changes to the institutional field or to the partnerships (Matos-Castaño et al. 2014).

Transformation has a very broad usage of the term, from transforming a product to transforming an industry to transforming an ecosystem. The next quotation shows transformation of the structures within a system. This is from an article about transformation of public health delivery system structures (Ingram et al. 2012).

Four key determinants of structural change emerged: availability of financial resources, interorganizational relationships, public health agency organization, and political relationships. Systems that had changed were more likely to experience strengthened partnerships between public health agencies and other community organizations and enjoy support from policy makers, while stable systems were more likely to be characterized by strong partnerships between public health agencies and other governmental bodies and less supportive relationships with policy makers. (Ingram et al. 2012, p. 208)

The next quotation on transformation uses a meaning somewhat similar to the last, focusing on the structure of the entities involved, but also adding content about the impact of this new structure as the goal. It is from an article on transformation in the wine industry (McDermott et al. 2009). This quotation provides a definition on what transformation means:

[the firm] has led this change, pioneering a new constellation of institutions and interfirm networks that appears to have facilitated widespread product up-grading. (McDermott et al. 2009, p. 1271)

Transformation is also used in the literature on social–ecological systems. From a negative perspective, it is the result of a change that impacted on a biophysical system or a social system in a way that is undesirable and likely irreversible (Smajgl et al. 2015). From a positive perspective, it is a change that significantly improves the situation. For example, from a climate perspective moving from a carbon-intensive economy to a low-carbon economy requires a transformation of many systems (Clarke and Ordonez-Ponce 2017). From the articles in our sample, the following quotation exemplifies this meaning:

Regions can be analyzed as dynamic and coupled social-ecological systems, which can vary in their ability to incorporate and adapt to change… This ability contributes to the resilience of a social-ecological system … Anthropogenic and biophysical influences can also transform a region, which implies a fundamental and potentially irreversible alteration of the system attributes and function … (Smajgl et al. 2015, p. 15)

For systemic change, this is focused on making major social or environmental improvements in a system, such as eradicating poverty or addressing unsustainable food supply (Senge et al. 2006). Another example of this term is the following quotation on child adoption systems, which talks about process improvements that will lead to improved impacts.

Most importantly, SWAN has achieved long lasting systemic changes, including an increase in the number of private agencies providing special needs adoption services, new standards for best practice in adoptions, and a network that collaboratively identifies solutions to emerging problems. (Jones 1999, p. 594)

While the search terms—institutional change, transformation, systemic change, and systems change—are hardly synonyms, they are used for similar meanings in many of the articles within our final sample of 100 (but different meanings in other articles we excluded). Transformation, in particular, also is used for other meanings such as change (with no relation to a system). Clearly though, there is no precise specification within the literature on what makes change “systemic” in the context of cross-sector partnerships.

Despite this lack of precision in the literature, the inferences discussed above suggest that there are a number of relatively common characteristics across the ways that systemic change is discussed in the literature, and that should form the basis for any definition going forward. Therefore, we propose that systemic change in the context of cross-sector partnerships should be defined as follows:

Systemic change: the result of actions that lead to a significant alteration within a system, potentially leading to substantial impacts. The system can be at any scale. Examples of systemic change include a fundamental change in policy, transformation of the structure in an institutional field, and significant change in system attributes or function.

**Fig. 2:**
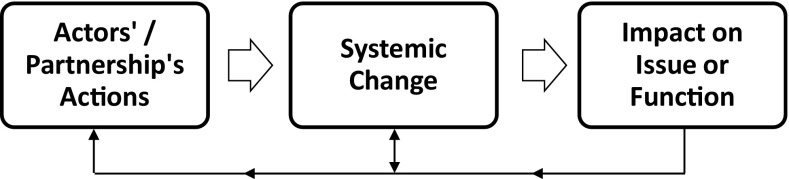
Systemic change

Figure 2 offers a depiction of systemic change that differentiates it from the actor’s actions and from the impact on the issue or function.

Studies on systemic change can focus on: the actors’ actions that lead to systemic change; the systemic change (and the role of actors); the impacts and the systemic changes that led to those impacts; the overarching issue and how it relates to any of these steps; the institutional environment and how it relates to any of these steps; or any number of other study boundaries. For this Thematic Symposium, the focus is in the first two boxes of Fig. 2 with some papers being more centred on the partnership’s actions and others more focused on the relationship between actors and systemic change, but all relate to cross-sector partnerships for systemic change. While our review did not distinguish between socially oriented partnerships and partnerships more generally, this Thematic Symposium is particularly interested in collaborative arrangements aiming for systemic change that is intended to lead to positive social, environmental, or economic impacts.

### Introducing the Thematic Symposium

The articles in the Thematic Symposium were all originally presented at the fifth biennial *International Symposium on Cross*-*Sector Social Interactions* (CSSI 2016) hosted by the University of Waterloo and York University in Toronto in April 2016. The theme of the CSSI 2016 conference, which subsequently became the title of this Thematic Symposium, was “Cross Sector Partnerships for Systemic Change”. Our goal in choosing this theme was to explore the potential and limits of cross-sector collaboration for forging deep-level change in social, economic, and/or environmental systems.

All papers presented at CSSI 2016 were eligible for submission to the Thematic Symposium, but in selecting those that went out for peer review, we prioritized those that directly addressed the theme. In all 12 papers were submitted, of which six were finally accepted following double-blind peer review.

As with the broader literature that we have reviewed above, the six papers that follow take a variety of perspectives on systemic change and the role of cross-sector partnerships in achieving such change. Here we introduce each by explaining briefly their approach and conclusions.

#### Relationships Between Cross-Sector Partnerships and Systemic Change

Half of the papers in the Thematic Symposium focus on the relationships between the partnerships and systemic change—bound by the issues or problems that actors are seeking to achieve change in. This, for example, is evident in the article by Van Tulder and Keen (2018) entitled “Capturing Collaborative Challenges: Designing Complexity-Sensitive Theories of Change for Cross-Sector Partnerships”. Here, the authors conceptualize systemic change in terms of change that occurs in relation to issues across sectors, or more broadly speaking, “complex” change. They argue that partnerships focused on such complex, systemic issues need to be configured differently to those aimed at relatively simpler issues. They offer a roadmap for designing such partnerships, centring on the development of a theory of change that is sensitive to the level of complexity involved.

Dentoni, Bitzer and Schouten (2018) in their article, “Harnessing Wicked Problems in Multi-Stakeholder Partnerships”, are also concerned with how change is effected in what they variously describe as “complex” or “wicked” problems. Like Van Tulder and Keen (2018), they are concerned with how to achieve “deep-level change” or “deeper processes of systemic change” in relation to wicked problems. In the article, they address cross-sector partnerships through the lens of “collaborative governance” (one of the search terms we used for our review), specifically in the context of multi-stakeholder initiatives designed to address complex problems. Interestingly, their analysis of the governance processes of the Roundtable on Sustainable Palm Oil focuses on two dimensions of systemic change: (1) the *depth* of systemic change, which they equate with changes in power structures; and (2) the *breadth* of systemic change, which they relate to changes in practices across sectors and spheres of action. This distinction is quite novel in the literature on cross-sector partnerships and systemic change and provides a useful platform for examining in slightly more sophisticated ways what we mean by some of these deeper-level objects of systemic change.

Quarshie and Leuschner (2018) in their article “Cross-Sector Social Interactions and Systemic Change in Disaster Response: A Qualitative Study” consider how the United States National Preparedness System has evolved as an example of systemic change. They study how the processes of cross-sector social interactions and systemic change interlink. The article offers a model to explain how cross-sector interactions (specifically the social mechanisms of learning, regulating, interconnecting, and re-engineering) lead to systemic change.

#### Actors as the Focus for Partnerships and Systemic Change

As we have already explained, systemic change in the context of cross-sector partnerships can also refer to the subjects of systemic change, namely the actors involved in partnerships and in the systems addressing social and environmental problems. Shumate et al. (2018) with their article on “Does Cross-Sector Collaboration Lead to Higher Nonprofit Capacity?” provide an example of a study that is almost entirely focused on the partnership actors box in Fig. 2. This study asks if cross-sector partnerships actually enable a non-profit organization to better contribute to systemic change (from the perspective of increased internal capacity). Through a large-scale quantitative study of 452 non-profit organizations, they showed that being involved in more cross-sector partnerships does not increase the organization’s capacity. That said, some types of enduring cross-sector partnerships improve strategic planning capacity within the organization. The boundary of this study is on the partner, and not the partnership or the issue to be addressed, all in the context of cross-sector partnerships for systemic change.

Klitsie et al.’s (2018) article “Maintenance of Cross-Sector Partnerships: The Role of Frames in Sustained Collaboration” focuses on a partnership as the unit of analysis, and within that partnership the role of framing mechanisms in ensuring successful collaboration. The study considers 8 years of a large cross-sector partnership (30+ partners) that exists to create a market for recycled phosphorus (a nutrient in crop growth) to address concerns about food security. The Nutrient Platform (the partnership) has been able to achieve significant regulatory reform (i.e. systemic change). The article considers how collaboration is sustained over time by allowing an optimal number of frames about the issue by a diverse array of partners. They argue that progress on agreements can be thwarted by too many frames.

Trujillo (2018), in her article “Multiparty Alliances and Systemic Change: the Role of Beneficiaries and their Capacity for Collective Action” focuses specifically on the effects of partnerships on beneficiaries and specifically on their capacity for collective action. She explores regional cross-sector partnerships in Colombia addressing poverty and violence but rather than conceptualizing poverty or violence as a complex, systemic problem, she examines how the partnerships lead to a transformation in the system of actors themselves around the issues. Drawing on a rich multiple case analysis, she identifies the processes through which beneficiaries can develop and acquire collective action capacity and how this enhanced capacity can in turn lead to increased potential for further transformative change.

## Conclusion

As is clear from our review of the literature and the articles in this Thematic Symposium, there is growing interest among scholars in the subject of systemic change within research on cross-sector partnerships. It is also evident that the debate is a transdisciplinary one, with research on these issues published in a wide variety of disciplines. Partly as a result of this, there is tremendous diversity in the types of system that are addressed, how they are defined, and their scale. Going forward, there is clearly much work to be done in developing this stream of literature and ensuring that it has meaningful scholarly and practical impact. We believe that, in particular, four key issues need to be addressed by future researchers in this space.

First, scholars interested in systemic change in the context of cross-sector partnerships need to pay much greater attention to defining what they mean by the term and developing clear constructs to conceptualize and operationalize systemic change for theoretical and empirical work. The lack of construct clarity to date potentially undermines the important work that is taking place exploring the broader changes sometimes associated with partnerships. It has been extremely difficult for researchers to build upon each other’s work because there is no clear sense of what it is they are actually examining and whether subsequent studies are exploring something similar or different. As Suddaby (2010, p. 347) explains, “constructs are the building blocks of strong theory”, and without better construct clarity, cross-sector research on systemic change runs the risk of continually proliferating without making meaningful theoretical advancement. We hope that our definition presented earlier will be helpful in this respect, even though we recognize that this is but a first attempt and that there is considerable scope for more fine-grained delineation of specific types and forms of systemic change.

Second, we believe that future research could and should better acknowledge and embrace the inherent interdisciplinarity in the field. When we seek to understand systems, we are typically required to deal with a range of different actors, activities, and impacts, many of which may not be the usual subjects of scholarly research in our discipline. For this reason, it behoves scholars of cross-sector partnerships interested in systemic change to consider the wide range of research that has been conducted on the subject, with a view to getting a clearer sense of what has already been accomplished and understood about the phenomena they are interested in and to prevent pointless replication. Again, hopefully our review will provide an initial insight into what some of this base of literature is, and where it can be found, but there is still a long way to go to build effective bridges across these disciplines.

Third, for management researchers in particular, there is understandably a considerable amount of attention on the actors and partnerships involved in seeking to achieve systemic change and how they interact with such change, not least because management research typically engages in research at the level of individual and especially organizational actors. However, there is considerable scope for new research that more explicitly also addresses the impact of systemic change on the issue itself. While work is underway linking cross-sector partnerships to different types of impact (e.g. Clarke 2011; Clarke and Ordonez-Ponce 2017; MacDonald et al. 2018; Van Tulder and Keen 2018; Van Tulder et al. 2015), the question of how we can address the interactions between different levels of actors, systems, and actual change for the issue itself remains a key challenge for the future.

Finally, a whole range of theoretical and methodological questions about how best to develop and design research that addresses systemic change remain. The articles in this Thematic Symposium include quantitative, qualitative, and conceptual research but with an emphasis on qualitative case study based work. This is typical of a business and society field in emergence (Crane et al. 2018), but it can also be associated with predominantly exploratory and descriptive research. The challenge for researchers in this field will be to develop more theoretically driven studies that connect with and extend extant work in new and important ways. While we may be a long way from developing an integrated theory of cross-sector partnerships for systemic change, future research should take greater account of the need to build on what is already known and to develop more sophisticated methodologies for identifying, interpreting, and ultimately measuring systemic change based on commonly agreed conceptualizations. Research on systemic change is alive and well among those interested in cross-sector partnerships but now is the time for concerted efforts to build a theoretically stronger, more integrated, and more methodologically advanced corpus of literature to help make sense of this critically important phenomenon.
